# Practical guidance for CD management involving treatment of botulinum toxin: a consensus statement

**DOI:** 10.1007/s00415-015-7703-x

**Published:** 2015-04-01

**Authors:** Alberto Albanese, Giovanni Abbruzzese, Dirk Dressler, Wojciech Duzynski, Svetlana Khatkova, Maria Jose Marti, Pablo Mir, Cesare Montecucco, Elena Moro, Michaela Pinter, Maja Relja, Emmanuel Roze, Inger Marie Skogseid, Sofiya Timerbaeva, Charalampos Tzoulis

**Affiliations:** 1 Neurologia I Istituto Neurologico Carlo Besta, Università Cattolica del Sacro Cuore, Via G. Celoria, 11, 20133 Milan, Italy; 2 Centre for Parkinson’s Disease and Movement Disorders DINOGMI, University of Genoa Largo Daneo 3, 16132 Genoa, Italy; 3 Movement Disorders Section, Department of Neurology, Hannover Medical School, Hannover, Germany; 4 Section of Neurology, Department of Clinical Sciences, Lund University, Jan Waldenströms gata 15, 205 02 Malmö, Sweden; 5 Neurological Department for Post-Stroke Patients, Moscow Federal State Hospital for Treatment and Rehabilitation Ministry of Health Russia, Moscow, Russia; 6 Neurology Service, Institut Cliníc de Neurosciències (ICN), CIBERNED, Hospital Clinic of Barcelona, Barcelona, Spain; 7 Unidad de Trastornos del Movimiento, Servicio de Neurología y Neurofisiología Clínica, Instituto de Biomedicina de Sevilla (IBiS), Hospital Universitario Virgen del Rocío/CSIC/Universidad de Sevilla, Seville, Spain; 8 Centro de Investigación Biomédica en Red sobre Enfermedades Neurodegenerativas (CIBERNED), Seville, Spain; 9 Department of Biomedical Sciences, University of Padova, Via Ugo Bassi n. 58/B, 35121 Padua, Italy; 10 Division of Neurology, CHU of Grenoble, Joseph Fourier University, Grenoble, France; 11 Center for Neurorehabilitation, Department for Clinical Neurosciences and Preventive Medicine, Danube University Krems, Dr. Karl-Dorrek-Straße 30, 3500 Krems, Austria; 12 Referral Center for Movement Disorders, Department of Neurology, School of Medicine, University Hospital Center Zagreb, University of Zagreb, Kispaticeva 12, 10000 Zagreb, Croatia; 13 AP-HP, Hôpital de la Pitié Salpêtrière, Département de Neurologie, 75013 Paris, France; 14 Inserm U 1127, CNRS UMR 7225, Sorbonne Universités, UPMC Univ Paris 06 UMR S 1127, Institut du Cerveau et de la Moelle épinière, ICM, 75013 Paris, France; 15 Movement Disorders Unit, Department of Neurology, Oslo University Hospital, Rikshospitalet, Oslo, Norway; 16 Department of Neurogenetics, Research Center of Neurology, Russian Academy of Medical Sciences, 80 Volokolamskoye shosse, Moscow, 125367 Russian Federation; 17 Department of Neurology, Haukeland University Hospital, Bergen, Norway; 18 Department of Clinical Medicine, University of Bergen, Bergen, Norway

**Keywords:** Dystonia, Torticollis, Botulinum toxins, Botulinum toxin type A, Decision making, Consensus development conference

## Abstract

Cervical dystonia is a neurological movement disorder causing abnormal posture of the head. It may be accompanied by involuntary movements which are sometimes tremulous. The condition has marked effects on patients’ self-image, and adversely affects quality of life, social relationships and employment. Botulinum neurotoxin (BoNT) is the treatment of choice for CD and its efficacy and safety have been extensively studied in clinical trials. However, current guidelines do not provide enough practical information for physicians who wish to use this valuable treatment in a real-life setting. In addition, patients and physicians may have different perceptions of what successful treatment outcomes should be. Consequently, an international group of expert neurologists, experienced in BoNT treatment, met to review the literature and pool their extensive clinical experience to give practical guidance about treatment of CD with BoNT. Eight topic headings were considered: the place of BoNT within CD treatment options; patient perspectives and desires for treatment; assessment and goal setting; starting treatment with BoNT-A; follow-up sessions; management of side effects; management of non-response; switching between different BoNT products. One rapporteur took responsibility for summarising the current literature for each topic, while the consensus statements were developed by the entire expert group. These statements are presented here along with a discussion of the background information.

## Introduction

Cervical dystonia (CD) is a movement disorder characterised by inappropriate contractions of the cervical musculature related to a dysfunction of sensorimotor neural circuits. It causes involuntary movements of the neck and head, which may be accompanied by tremor, and results in abnormal postures. In addition to the impaired neck mobility and abnormal posturing, chronic or frequently occurring neck pain is a recognised clinical feature of CD that occurs more frequently than in other forms of dystonia [1]. This constellation of symptoms, and a reduction in the patient’s self-image, may result in disability and adversely impact the individual’s quality of life [2, 3].

Botulinum neurotoxin (BoNT) treatment is the accepted standard of care for patients with CD and the preferred toxin type is BoNT-A. There are a number of published guidelines which deal with the general aspects, but do not cover the many practical variables that influence outcome of BoNT treatment for CD [4–6]. The success of treatment is very dependent on the experience and ability of the injector, both to identify and to treat the involved muscles, yet there is little practical guidance available to help the treating physician achieve optimal results. Additionally, as experienced injectors, we have observed that patients have different individual needs and expectations of treatment. There may also be different perceptions between the patient and the treating physician on what should be the goals of treatment and what is deemed a satisfactory outcome. Therefore, an international group of neurologists, experts in this area, were brought together to produce consensus statements about the key practical issues that can contribute to achieving the highest degree of patient satisfaction with BoNT-A treatment in CD.

## Methods

An International Consensus Committee, consisting of 15 neurologists with extensive experience in the treatment of CD with BoNT, was established to review the literature and provide a consensus. The preparatory work of the group consisted of a search and review of pertinent publications on the evaluation and treatment of cervical dystonia, with particular regard to patient perspectives and treatment with BoNT-A. Computerised MEDLINE searches including publications from 2007 to 2013 were conducted using a combination of text words and MeSH terms: [“torticollis”(MESH terms) OR “torticollis”(all fields) OR “cervical”(all fields) AND “dystonia”(all fields) OR “cervical dystonia”(all fields)] and limited to human studies. The reference lists of all known primary articles were searched for additional, relevant citations, which were also included even if they were published before 2007. No language restrictions were applied.

The topic was divided into eight sections that were chosen to address the practicalities of real-life treatment of CD: (1) place of BoNT within CD treatment options, (2) patient perspectives and desire for treatment, (3) assessment and goal setting, (4) starting treatment with BoNT-A, (5) follow-up sessions, (6) management of side effects, (7) management of non-response and (8) switching between different BoNT products. The search results were then reviewed to locate the papers relevant to each section.

Eight rapporteurs took responsibility for one section each and developed a presentation to be discussed within the entire group during a 1-day meeting, from which the consensus statements were developed. The group utilised the consensus development conference methodology to provide consensus statements on the use of BoNT-A in CD [7].

Although several formulations of BoNT are available, this consensus focuses on the most widely investigated and widely used preparations of botulinum toxin A: onabotulinumtoxinA (BoNT-A/Ona, Botox^®^ Allergan); abobotulinumtoxinA (BoNT-A/Abo, Dysport^®^ Ipsen) and incobotulinumtoxinA (BoNT-A/Inco, Xeomin^®^ Merz). RimabotulinumtoxinB (BoNT-B/Rima, Myobloc^®^ USWorldMeds, Neurobloc^®^, Eisai) was also mentioned.

## Results

### Consensus statements, key literature and commentary

The consensus statements are summarised in Table 1.

**Table 1:** Consensus statements from the international group of experts

Statements	Key literature, selected clinical studies and reviews
Place of BoNT within CD treatment options	
BoNT-A is the first-line standard of care for cervical dystonia There is Level A evidence for its efficacy and safety All formulations of BoNT-A have similar indications for CD	Class I^a^ placebo-controlled studies: [48, 52, 88–92] Class I comparator studies: [74, 80, 91–93]
Patient perspectives and expectations from treatment	
Patients’ opinions are essential in shaping treatment objectives and goals BoNT treatment can improve health-related quality of life in patients with CD There may be a difference between the patient’s perception and the physician’s judgement that might require adequate discussions between the patient and doctor The patient’s perception may be influenced by non-motor factors and/or comorbidities (e.g. psychiatric and emotional) Appropriate interaction between the patient and the doctor will lead to optimal treatment planning	Class II: [2, 28, 29] Class III: [20, 21, 27, 30, 31] Review: [24]
Assessment and goal setting	
Accurate clinical diagnosis is essential and pretreatment assessment is essential Several scales are available for CD Rating scales can be used at baseline and periodically to assess the patient’s condition over time The Movement Disorders Taskforce recommends TWSTRS, CDIP and CDQ-24 None of these scales was constructed to influence treatment with BoNT (Dystonia Discomfort Scale may give more information on BoNT therapy) It may be useful to conduct clinical evaluation 4–6 weeks after first treatment or unsatisfactory response	[36]
Starting treatment with BoNT-A	
BoNT-A is the treatment of choice (BoNT-B is reserved for patients with resistance to BoNT-A) Correct muscle selection is paramount for treatment’s success BoNT therapy should be initiated in the primarily affected muscles Dosing must be tailored to the individual patient based on the patient’s head and neck position, location of pain, muscle hypertrophy and muscle size Initial dosing in a naïve patient should begin at a dose in the lower range For each muscle consider optimal concentration, number of units and number of injections This information should be recorded PT might be useful combined with BoNT treatment (insufficient evidence)	[38, 39] [9, 46, 47]
Follow-up sessions	
Reinjections should not be performed until efficacy starts declining Muscle selection, dose and injection interval should be adjusted accordingly There is no robust evidence to support a fixed reinjection interval of 12 weeks Clinical evidence indicates that CD patients may be better treated using individually adjusted intervals In a controlled clinical study, the shortest injection interval was 6 weeks. The range extended to 33 weeks. Consider the possibility of remission Follow-up sessions should review the patient’s response to the previous injections with regard to Clinical response Subjective response Functioning	[31, 49–52]
Management of adverse events	
Precision in placing injections is necessary to prevent unwanted spread of toxin and adverse events Administer an appropriate dose in each muscle and modify the injection scheme based on the patient’s response, balancing the efficacy and side effects There is no evidence suggesting differences in side effect profiles between marketed BoNT-A formulations	[54–56] [57–60]
Management of non-response	
Primary non-response Definition: there is subjective and objective evidence of no change in motor pattern following at least 3 consecutive cycles of appropriate treatment in a treatment-naive patient Reconsider the diagnosis of CD Perform an EDB or frontalis test to verify if there is a biological response to BoNT-A Positive test (no paralysis): shift to another BoNT Negative test: reconsider the treatment strategy; then consider other therapeutic approaches Review muscle selection and doses Secondary non-response Definition of non-response: there is subjective and objective evidence of no change in motor pattern following at least 2 consecutive cycles of appropriate treatment in a patient who was previously responding to treatment Reinjection should not be performed to potentiate a previous treatment, particularly in cases of non-response Perform an EDB or frontalis test to verify if there is a biological response to BoNT Positive test (no paralysis): consider other options such as switching BoNT formulation or proposing DBS Negative test: reconsider the treatment strategy; then consider other therapeutic approaches	[62]
Switching between different BoTN products	
Physicians need to be familiar with each individual type of toxin they use Conversion between BoNTs provides some challenges and should be undertaken with caution Switching between Botox and Xeomin is most straightforward with a 1:1 conversion ratio Switching Botox to/from Dysport or Neurobloc is more complex as there is no clear conversion ratio	[69–73] [59, 65, 73, 79–84]

^a^Class ascribed by two reviewers using American Academy of Neurology (AAN) classification (from reference [94])

#### Place of BoNT within CD treatment options—Rapporteur Maja Relja

Initially, cervical dystonia was treated with oral medications, or surgical interventions, but their effects were disappointing in most cases [8, 9]. Consequently, chemodenervation using BoNT-A has become the cornerstone of treatment for CD with a good safety and efficacy profile. However, surgical treatment, particularly pallidal neurostimulation, may still be considered for patients with severe CD refractory to the combination of oral drugs and chemodenervation [10, 11]. Adjuvant physiotherapy might be proposed regardless of the therapeutic option.

Oral treatment (including anticholinergic agents, γ-aminobutyric acid (GABA) mimetic agents, dopamine receptor antagonists, dopamine-depleting agents and even dopamine receptor agonists) is limited in efficacy and there is a lack of sound scientific evidence supporting the use of most agents [4]. Systemic effects of oral therapy are non-selective, often causing generalised and problematic adverse events. The many different treatments tried over the years also testify to both their relative ineffectiveness and the recalcitrant nature of cervical dystonia. Once the efficacy of BoNT had been demonstrated in the 1980s, it rapidly assumed a place as the treatment of choice for this condition, warranting both European Federation of Neurological Societies EFNS and the American Association of Neurology (AAN) level A recommendations as first-line treatment [5, 6, 12, 13].

A recent systematic review of the current literature has supported this position, showing that BoNT is the most effective treatment for reducing dystonic symptoms and pain in patients with focal dystonia [14].

BoNT treatment has a peak effect few weeks after treatment and tends to decay variably over time. Given the chronic nature and the varying features of dystonia over time, changes in dosage and targeting may be required over repeated treatment sessions. The amplitude of the response to BoNT-A has been investigated in terms of reduction of the maximal voluntary EMG amplitude in the sternocleidomastoid muscle in a group of 34 patients with cervical dystonia undergoing regular BoNT-A therapy with BoNT-A/Ona (*n* = 16) or BoNT-A/Abo [15]. Dose-dependent EMG amplitude reductions were seen with both toxin preparations: with BoNT-A/Ona this ranged from 80 to 91 % in response to 20–80 units; with BoNT-A/Abo the responses ranged from 80 to 91 % with doses of 100–500 units.

The efficacy of BoNT-A has been shown to be sustained over at least 12 years (mean 15.8 ± 1.5 years) of continuous use [16] in a longitudinal follow-up study. Two reviews have also addressed the long-term efficacy and safety of botulinum toxin [17, 18]. These authors concluded that the majority of patients comply with long-term treatment because they experience positive and stable effects over time, and there is no evidence of specific adverse events through the long-term use of botulinum toxins.

#### Patient perspectives and expectations from treatment—–Rapporteur Inger Marie Skogseid

Alongside the clearly visible dystonic symptoms of abnormal movements or postures, patients with CD often experience functional disability, pain and other sensory disturbances including impaired proprioception [19–23] and sometimes depression and/or anxiety. Increasingly, these symptoms are being termed non-motor [24], although the term is not universally accepted, because in many patients these effects are probably, at least in part, secondary to the motor symptoms. These symptoms can cause reduced ability to work [19, 21, 25, 26], impaired social functioning and social stigma [26, 27] and sometimes impair activities of daily living, including those of personal hygiene. These factors (especially pain), which impact on employment and psychosocial functioning, are the most common reasons for patients seeking treatment.

The marked negative impact of CD on patients’ quality of life [2] can be ameliorated by effective BoNT treatment [27–29]. The majority of studies used established QoL instruments, the most popular of which was the Short Form-36 Health Survey (SF-36) [2, 27, 28, 30]. All of these showed decreased HRQoL in patients with CD compared with healthy volunteers (either control groups in the study or by comparison with the general population).

BoNT-A treatment improved several domains of the SF-36. Interestingly, the improvement seen on patient-related QoL parameters was not always correlated with physician assessments of the effect of BoNT-A using measures such as the TWSTRS score. Using a combination of patient-reported and physician-reported scores, Skogseid et al. [27] showed that the majority of the CD population undergoing long-term treatment with BoNT-A achieved a good HRQoL. Those with poorer HRQoL scores tended to be those with higher TWSTRS scores and greater degrees of depression.

Effective treatment can also improve their employment status and decrease depression among CD patients, which is one of the most important predictive factors for poor quality of life [21, 27, 28, 30].

At the outset of treatment it is essential to discuss with the patients what they can expect from the treatment and to ensure that there is sufficient understanding between patients and physicians about the treatment goals they have and which are achievable and realistic. Indeed, studies have shown that patients’ perceptions and neurologists’ perceptions of treatment satisfaction are not always aligned [20, 31].

#### Assessment and goal setting—Rapporteur Charalampos Tzoulis

Assessment of patients with CD is aimed at identifying the clinical features and aetiology based on the current classification [32] and rating motor and non-motor clinical features as a basis for follow-up.

The cornerstone of diagnosis is the clinical examination of abnormal involuntary movements and postures [26, 33]. Currently, there is no consensus on diagnostic criteria for CD and misdiagnosis is not a rare occurrence [34]. A recent consensus update considered two axes for classification: it provided a snapshot of the patient’s clinical features that can be repeated in time for comparison, on Axis I, and an aetiological classification that is reassessed when needed, on Axis II [32].

Patient assessment prior to injection should consider the clinical aspects that may influence the selection of muscles for injection. Patients may use compensatory movements to overcome forceful dystonic posturing, such as neck flexion, or trunk anteroflexion to overcome a retrocollis, and such movements must be distinguished from primary CD components [35]. Another aspect is muscle weakness or atrophy that may be secondary to previous BoNT treatment. Muscles involved in compensatory movements and weak muscles are usually not injected with BoNT. Pain may be caused by muscle contraction, in which case it provides a useful orientation for muscle targeting, or by overstretching of opponent muscles. Chronic pain may be sustained by local release of pain mediators or musculotendinous inflammation.

Dystonic tremor may respond less well than dystonic postures to BoNT treatment. Usually, postural abnormalities and pain are first-line criteria for muscle choice.

Rating of CD is commonly performed using dedicated scales. Scoring at baseline, at the time of peak effect (approximately, 1 month after injection) and before retreatment, allows injectors to assess outcome and plan changes in dosing and targeting, if necessary. The most commonly used dystonia scales have been the object of a recent revision by a Movement Disorders Society task force [36]. The Toronto Western Spasmodic Torticollis Rating Scale (TWSTRS), the Cervical Dystonia Impact Scale (CDIP-58) and the Cervical Dystonia Questionnaire (CDQ-24) are “recommended” for cervical dystonia; the Functional Disability Questionnaire, the Tsui Scale and the Body Concept Scale have been rated as “suggested”. Of these, a physician-rated severity score is found in the TWSTRS and the Tsui scores. Recently, the Dystonia Discomfort Scale (DDS) has been introduced as a novel instrument to monitor the temporal profile of BoNT therapy in CD patients who can perform a self-assessment at home [37]. Furthermore, a new version of the TWSTRS is currently being validated in North America and Europe.

#### Starting treatment with BoNT-A—Rapporteur Elena Moro

The main goal of treating CD with BoNT is to correct both dystonic movements and abnormal postures and thus be able to reduce pain, discomfort, and functional disability and improve patient’s quality of life.

When a CD patient undergoes the initial BoNT treatment, the first step is muscle selection. This is a crucial step and relies on accurate clinical examination of the patient.

Dystonic malposture may affect only the cervical spine itself (-collis, 20 % of cases) or only the head´s position in relation to the cervical spine (-caput, 19 % of cases) or a combination of both (61 % of cases). A detailed classification of the different abnormal postures in CD patients has been recently proposed by Reichel [38]. By understanding this classification, injection of BoNT into muscles that are not involved can be avoided. As well as the anatomical location, it is important to understand the function of the involved muscles. The dystonic posture should be analysed in a 3D space and using activation and deactivation tasks.

Dystonic muscle activity may be mainly tonic, myoclonic, tremulous or a complex mixture. In simple cases, an accurate clinical examination will usually allow identification of the primary involved muscles and enable them to be separated from muscles with compensatory activity. Faster dystonic movements also need to be assessed, since their response to BoNT is not so predictable. Although clinical examination allows the evaluation of all motor components of CD, for more complex or unclear cases electromyography (EMG) mapping can be very useful for a more accurate selection of the most relevant and active dystonic muscles.

A recent study has further detailed the clinical phenotypes of dystonic posterior sagittal shift or “double chin” posture and anterior sagittal shift or “goose neck” posture. They used clinical examination and EMG to define more clearly the involved muscles and proposed specific BoNT treatment protocols for these forms of CD [39]. A new technique of injection of the longus colli, based on a laterocervical approach under EMG guidance, has also been described.

Injections may be carried out using visual inspection and palpation of muscles for the more superficial muscles; EMG or ultrasound is recommended for accurately locating deeper muscles [40, 41]. A summary of how to conduct muscle identification is given in Table 2.

**Table 2:** Clinical examination in CD patients

For simple cases, clinical examination allows the identification of the primarily involved muscles, as opposed to compensatory activity
Examine postures and movements in a 3D space plane
At rest in a sitting position (also with eyes closed)
Using activation and deactivation tasks (walking, standing, active resistance, finger-nose test, arm-hold test, etc.)
Consider in which direction the head/neck moves spontaneously and easily, and in which direction there is resistance/reduced range of motion
Consider *geste antagoniste*/trick manoeuvre
Consider muscle hypertrophy
Consider pain, contraction and tenderness on palpation
As a rule of thumb, the muscles that cause the most dominant movement of head/neck and that are clinically hyperactive and painful should be injected
For more complex or unclear cases, the use of EMG mapping can be useful
EMG and ultrasound can be very useful for injecting deep muscles
Consider videotaping the clinical examination before injections

The second step consists of choosing which BoNT formulation to use. All marketed BoNT brands are licensed for cervical dystonia [42–45] and can be used for treatment.

The third step concerns the use of the appropriate BoNT dose and dilution. Doses must be tailored to the individual patient, based on type of muscle, the degree of muscle activity, muscle size, and sometimes location of pain. There are different minimal starting doses suggested for each muscle. Dose recommendations by muscle are given in Table 3 (modified from [46] ).The concentration of toxin, number of units and number of injections/muscle should be recorded for each muscle.

**Table 3:** Muscles commonly affected in cervical dystonia, their function, and BoNT doses currently used (from reference [46])

Muscle name	Function	BoNT-A/Ona/Inco (U)	BoNT-A/Abo (U)	BoNT-B/Rima (U)
Anterior muscles				
Longus collis	Flexion (forward)	15–30	20–60	N/A
	Mild rotation (ipsi)			
Longus capitis	Flexion (forward)	5–15	20–60	N/A
	Rotation (ipsi)			
Rectus capitis anterior	Flexion (forward)	2.5–10	10–30	N/A
Sternocleidomastoid	Rotation (contra)	20–50	40–120	1000–3000
	Tilt (ipsi)			
	Sagittal shift (backward)			
	Flexion (forward)			
Lateral muscles				
Anterior scalene	Tilt (ipsi)	5–30	20–100	500–2000
	Rotation (contra)			
	Flexion (forward)			
Middle scalene	Tilt (ipsi)	5–30	20–100	500–2000
	Rotation (contra)			
Rectus capitis lateralis	Tilt (ipsi)	N/A	N/A	N/A
Posterior scalene	Tilt (ipsi)	5–30	20–100	500–2000
	Mild rotation (contra)			
Posterior muscles				
Splenius capitis	Rotation (ipsi)	40–100	100–350	1000–4000
	Tilt (ipsi)			
	Sagittal shift (backward)			
	Extension (backward)			
Semispinalis capitis	Rotation (contra)	20–100	60–250	1000–2000
	Tilt (ipsi)			
	Extension (backward)			
Trapezius	Shoulder elevation	25–100	60–300	1000–4000
	Extension (backward)			
	Sagittal shift (backward)			
	Tilt (ipsi)			
	Rotation (assists in ipsi and contra)			
Levator scapulae	Shoulder and scapula elevation	20–100	60–200	1000–2000
	Tilt (ipsi)			
	Rotation (contra)			
Obliquus capitis inferior	Rotation (ipsi)	10–20	50–80	N/A
Rectus capitis posterior	Rotation (ipsi)	2.5–10	10–30	N/A

The distinction into anterior, lateral, and posterior muscles is aimed at providing a schematic distinction, as several muscles produce multiple movements. First-treatment doses should not exceed 200 BoNT-A/Ona/Inco U, 500 BoNT-A/Abo or 5000 BoNT-B/Rima U. Total dose/session should not exceed 400 BoNT-A/Ona/Inco U, 1000 BoNT-A/Abo U or 10,000 BoNT-B/Rima U

*N/A* evidence or personal experience not available

Patients should be informed about possible side effects and action profile of BoNT. They should be told that results may not be immediate after the first injection and that it normally takes up to about a week before the clinical effects of BoNT-A start to appear and then several days (or even 1–2 weeks) to reach its maximal effect. Importantly, patients should also know that titration of doses to reduce muscle activity over two to three treatment sessions may be necessary to achieve a significant symptom reduction and functional benefit.

It has been proposed that physical therapy can potentiate the effect of BoNT [9]. However, a systematic literature search has concluded that cautious interpretation on the effectiveness of physiotherapy as an adjuvant therapy is warranted, and that additional high-quality clinical trials are needed before firm conclusions can be drawn [47].

#### Follow-up sessions—Rapporteur Alberto Albanese

The majority of patients with CD require long-term treatment, involving regular, repeated injections. Patients should be assessed for their response to the initial treatment and subsequent injections based on: (a) clinical evidence of magnitude of response; (b) consideration of whether other muscles should be included; (c) patient perception of efficacy and duration of response; (d) severity and duration of side effects.

The current manufacturers’ literature suggests that the minimum period before repeating the treatment should be 10–12 weeks (Botox^®^ SmPC; Dysport^®^ SmPC; Xeomin^®^ SmPC) [42–44]. However, the original recommendation (which most subsequent trials followed) was based on very few patients (*n* = 28) and outcomes, and was using the original formulation of BoNT-A marketed by Allergan which carried a higher risk of developing immunoresistance due to its higher protein load [48].

Increasingly, patients and physicians find that an injection schedule with fixed intervals of 12 weeks may not be appropriate for all patients. Thus, the variability in duration of response to BoNT was studied in 404 patients across 38 centres [49]. Only 49.3 % of patients rated duration of response ≥ 12 weeks for all BoNT-A preparations. A further study assessed treatment duration (TD) (time between injection and patient-reported waning of effect) in 59 patients and showed that the mean TD/patient was 7.8 ± 1.4 to 21.0 ± 3.9 weeks [50]. A patient satisfaction survey conducted in patients treated with BoNT-A/Ona or BoNT-A/Abo has also shown that patient satisfaction with treatment declines prior to re-injection, and many (46 %) patients would prefer an injection schedule of less than 12 weeks [31].

Subsequently Evidente et al. [51] conducted a double-blind, randomised controlled trial of BoNT-A/Inco versus placebo in pre-treated or treatment-naive CD patients. A flexible dosing schedule was evaluated over a 68 week extension to the initial study of pre-treated or treatment-naive patients [52]. In those who received ≥ 2 injections, the median intervals were: 6 to ≤ 10 weeks in 22.5 %; > 10 to ≤ 12 weeks in 24.6 %; > 12 to ≥ 14 weeks in 19.4 % and > 14 weeks in 33.5 %.

This suggests that it might be useful to adopt a flexible dosing schedule. Flexible schedules may require some service adjustment to accommodate, but not necessarily an increase in the number of injections delivered overall, since the mean injection interval seen for these ‘flexible’ patients was still 12 weeks.

Dosing and targeting are usually varied during the first two to three treatment sessions and tend to stabilise afterwards, although adjustments also may be necessary at later sessions. Typically, the doses used in the first session are increased in some or all injected muscles and new muscles are considered for injection based on the remaining symptoms. To make these decisions, it is useful to have a patient assessment around 1 month after the preceding session, when BoNT action has peaked, and to compare it with the observed pattern of dystonia at the time of injection.

When considering dosing intervals, it is worth noting that in the study by Evidente et al. [51] in which flexible dosing intervals were employed, there were no differences in the tolerability profile between groups of patients injected at 6 to <10 weeks, 10 to ≤12 weeks or 12 to ≤14 weeks or >14 weeks and there were no instances of antibody formation.

#### Management of adverse events—Rapporteur Fina Marti

The adverse events of BoNT-A treatment are usually mild and self-limiting and similar in both nature and severity between the different formulations. A meta-analysis of 36 randomised controlled studies reported adverse events in 25 % (353/1425) of the BoNT-A (BoNT-A/Ona)-treated patient versus 15 % (133/884) in controls [53].

The most common adverse events related to BoNT-A are: dysphagia; neck muscle weakness; injection site pain; and ‘flu-like’ symptoms [18]. Adverse events of BoNT-A are dose related and mostly due to contiguous or distant spread of toxin. Therefore, it is important that injections are located precisely so that potential spread of toxin is minimised. Spread or diffusion of toxin into neighbouring muscles may be influenced by the injection technique, the dose employed, the concentration and also the volume of injection. Targeting can be improved by EMG- or ultrasound-guided placement of injections [41]. However, there is no agreement whether single or multiple injections should be placed in each muscle.

The chemodenervation achieved by the toxin is also dose dependent [54, 55]. It has been reported that a fivefold increase in toxin injection volume, but keeping the same dose of toxin, resulted in a 50 % increase in the affected area [56].

Using a hind limb muscle animal model, no differences were observed between different formulations of toxin (BoNT-A/Ona, BoNT-A/Abo or BoNT-A/Inco) [57] and diffusion was not affected by the molecular size of the toxin preparation and the presence of complexing proteins [58, 59]. Since all formulations have similar diffusion profiles, no specific advantages can be proposed for any particular formulation for treating the largest or smallest muscles involved in dystonia.

Recommendations for minimising adverse events include always using the lowest effective dose. Dysphagia can be reduced by giving sternocleidomastoid injections into the upper third of the muscle, increasing the concentration of toxin and by reducing the dose per muscle when giving bilateral sternocleidomastoid and hyoid muscle injections. For bilateral injections to the splenius capitis and semispinalis capitis, the individual muscle dose should also be reduced and a lower dose is advisable for any muscle weakened by previous treatment [60].

There are no adequate data from the use of botulinum toxin type A in pregnant women [61]. Studies in animals have shown reproductive toxicity. The potential risk for humans is unknown. The SPC for Botox^®^ does not recommend use during pregnancy and in women of child-bearing potential not using contraception [42]. Dysport^®^ should be used during pregnancy only if the benefit justifies any potential risk to the fetus [43]; Xeomin^®^ is contraindicated, unless the potential benefit justifies the risk [44].

#### Management of non-response—Rapporteur Giovanni Abbruzzese

Primary non-responders are those patients who do not get benefit from the first treatment. When this occurs the physician should first review the diagnosis and confirm that the patient’s condition is actually due to dystonia.

If the diagnosis of dystonia is robust and reliable, the most obvious cause of non-response is insufficient dosage or wrong muscle selection (this may make it difficult in some cases to distinguish between non-response and insufficient response). As mentioned previously, the lowest effective dose should be used at the outset of treatment to limit the risk of adverse events and may take two to three sessions before BoNT treatment reaches it maximal potential, during which time doses may need to be adjusted and refined. Current treatment recommendations for CD have reduced the frequency of non-response to 2 % over a treatment period of 2 years [62].

Secondary non-responders are those patients who fail to benefit at some point in time, having reported adequate benefit from previous treatment sessions. There is no universally accepted definition of secondary non-response to BoNT in CD.

An insufficient improvement in posture after  ≥3 unsuccessful injection cycles has been proposed as a defining criterion [63, 64]. It was originally thought that secondary non-response was due in the majority of cases to the presence of neutralising antibodies. Antibody formation is more likely when the protein load is high. Direct comparisons in an animal model showed differences in the potential for development of neutralising antibodies. After repeated intradermal injections in New Zealand white rabbits (16 U/animal for 8 administrations every 2–8 weeks for BoNT-A/Ona and BoNT-A/Inco; 40U/kg for 5 administrations over 13 week BoNT-A/Abo), the results showed that 15 rabbits developed antibodies after six injections of BoNT-A/Abo, 4 developed antibodies after nine injections of BoNT-A/Ona and none developed antibodies after BoNT-A/Inco [59, 65]. Formulations with low antigenicity may offer advantages when using high doses or if a dosing schedule with short interval is preferred for a particular patient. Most physicians would use the frontalis test or the extensor digitorum brevis test for detecting the presence of neutralising antibodies [66].

Exact and quantitative measurement of BoNT antibodies, however, is only possible by the mouse lethality test or the mouse diaphragm assay. A novel ELISA test, recently introduced, offers low-cost animal-friendly and sensitive BoNT antibody testing [67].

Another important cause of secondary non-response is insufficient dosing or inappropriate muscle selection, which can occur particularly if a fixed treatment scheme is repeated—without review—in subsequent treatment sessions [4, 60].

When secondary (or primary) non-response to BoNT-A (and BoNT-B) treatment persists, despite careful re-evaluation of both diagnosis and treatment by experienced dystonia specialists and injectors, deep brain stimulation (DBS) is a treatment option that is now under scrutiny [10, 11, 68].

#### Switching between different BoNT products—Rapporteur Emmanuel Roze

While it is usually considered desirable to maintain treatment with a formulation of BoNT-A that produces results judged satisfactory by both patient and physician, sometimes product switches may be required. The main causes for product switching include: non-response, changes in tenders to hospitals which may result in restricted product availability, health insurance restrictions, physician preference/experience, patient preference, when the patient requires different injection intervals and other individual needs.

Before 2005, only two formulations of BoNT-A were available: BoNT-A/Ona and BoNT-A/Abo, so this was the only product switch within BoNT-A usage that was relevant. With the introduction of BoNT-A/Inco, the scope for product switching has increased.

Considering BoNT-A/Ona to BoNT-A/Abo switch, studies showed highly variable results: the potency of BoNT-A/Ona relative to BoNT-A/Abo has been estimated to be 1:2 up to 1:11 [69, 70]. A randomised controlled trial later suggested a ratio of 1:3, but the products are still not equivalent at this ratio [71] and recent studies suggest that 1:4 may be a more appropriate ratio [72, 73]. These highly variable data led the manufacturers to warn against using a simple formula to convert dosages, since there is no simple dose equivalence. Consequently, when switching between BoNT-A/Ona and BoNT-A/Abo, it is advisable to gradually reduce the dose of BoNT-A/Ona, switch to BoNT-A/Abo and then to titrate the dose upwards, observing the patient’s response.

Where product switches have been achieved successfully, comparable efficacy can be achieved, although the doses are different. Odergren et al. [74] showed no statistical difference in Tsui scores comparing BoNT-A/Ona and BoNT-A/Abo (ratio 1:3), while clinical efficacy was better with BoNT-A/Ona compared with BoNT-A/Abo using TWSTRS (ratio 1:4 or 1:5 BoNT-A/Ona:BoNT-A/Abo) [75]. Better efficacy for BoNT-A/Abo compared with BoNT-A/Ona based on Tsui and TWSTRS (ratio 1:3 or 1:4 BoNT-A/Ona:BoNT-A/Abo) was instead reported by Ranoux et al. [76].

In comparing safety, a literature survey analysed 70 published articles in CD: 30 used BoNT-A/Ona, 24 used BoNT-A/Abo, 3 used BoNT-A/Ona + BoNT-A/Abo, 11 used BoNT-B/Rima and 2 used B-BoNT-A/Ona + BoNT-B/Rima. The mean total doses per treatment ranged from 60 to 374 U for BoNT-A/Ona, 125 to 1200 U for BoNT-A/Abo and 579 to 19,853 U for BoNT-B/Rima [77]. BoNT-A/Ona was associated with a significantly lower rate of dysphagia than BoNT-A/Abo: 10.5 % for original Botox (original 79-11 lot), 8.9 % for BoNT-A/Ona (current Botox) and 26.8 % for BoNT-A/Abo (both, *P* < 0.05). BoNT-B/Rima was associated with dry mouth (3.2–90.0 %) in 9 of 13 studies, but this effect was not reported in a sufficient number of BoNT-A studies (BoNT-A/Ona, *n* = 2; BoNT-A/Abo, *n* = 6) to permit statistical comparison.

Studies of the equivalence between BoNT-A/Ona to BoNT-A/Inco have produced much more consistent results. One preclinical study showed some minor differences [78], but these differences may be attributable to assay methodology. A more recent study has demonstrated equivalence between the two formulations [50]. In clinical evaluations, the equivalence of these two products has been reliably and repeatedly demonstrated [50, 59, 65, 73, 79–84].

BoNT-A/Ona and BoNT-A/Inco have been shown to have comparable efficacy and tolerability in healthy volunteers [79] and to have a similar duration of action in CD which does not show any ‘wearing off’ in up to 66 cycles of use [65, 73]. The ease of product switch, using a dose conversion ratio of 1:1, was demonstrated in a study of 263 patients, treated with BoNT-A/Ona for at least 1 year under stable conditions who were then converted to BoNT-A/Inco. After 3 years’ treatment, no subjective or objective differences were observed between BoNT-A/Ona and BoNT-A/Inco with respect to onset latency, maximum and duration of effect or adverse event profiles and there were no detectable differences in diffusion [73, 82].

When non-response is an issue, the treating physician should consider whether insufficient dosing or inappropriate or incomplete muscle selection are responsible [4]. If these approaches are not successful, then switching from BoNT-A to BoNT-B should be considered.

BoNT-A to BoNT-B switching is not a simple matter. There are large variations in conversion factors depending on the system used to compare the two toxin subtypes. The brow-furrow test suggests that conversion ratios of 50:1 or 100:1 (B:A) are effective—the latter producing a longer-lasting effect [85]; spasmodic dysphonia 52.3:1 [86]; skin model (29:1) [87]. Therefore, a practical approach is to start the new toxin at a lower dose than would be expected from conversion calculations (which are inaccurate) and then titrate upwards in relation to the patient’s response.

## Conclusion

CD is a neurological movement disorder that can affect both posture and movement of the head and neck, sometimes involving the shoulders. CD has varied and complex phenomenology, involving different muscles and combinations of muscles, more superficial or deeper, which may be affected in varying degrees and show different patterns of contraction—tonic, spasmodic, tremulous or a complex mixture. Treatment of CD should thus be tailored to the patients’ specific needs. BoNT-A is the first treatment of choice for CD. The aim of this publication has been to try and supply a consensus expert opinion on practical aspects of treatment for injectors to assist them in obtaining the highest degree of treatment satisfaction for their patients.

## Abbreviations

BoNT: Botulinum neurotoxin
BoNT-A: Botulinum neurotoxin type A
BoNT-B: Botulinum neurotoxin type B
CD: Cervical dystonia
HRQoL: Health related quality of life
QoL: Quality of life
